# Investigating the Development of Colorectal Cancer Based on Spatial Transcriptomics

**DOI:** 10.3390/ijms26189256

**Published:** 2025-09-22

**Authors:** Zhaoyao Qi, Guoqing Gu, Huanwei Huang, Beile Lyu, Yibo Liu, Wei Wang, Xu Zha, Xicheng Liu

**Affiliations:** 1 Laboratory for Clinical Medicine, Beijing Key Laboratory for Tumor Invasion and Metastasis, Department of Physiology and Pathophysiology, School of Basic Medical Sciences, Capital Medical University, Beijing 100069, China; 2 Laboratory for Clinical Medicine, Department of Physiology and Pathophysiology, School of Basic Medical Sciences, Capital Medical University, Beijing 100069, China

**Keywords:** colorectal cancer, spatial transcriptomics, pseudo-time analysis, enrichment analysis, bioinformatics

## Abstract

Colorectal cancer (CRC) remains a leading cause of cancer-related mortality worldwide. However, the spatial and temporal dynamics underlying its development remain poorly characterized. This study employs spatial transcriptomics (ST) to investigate the progression of intestinal tumors in *APC ^Min/+^* mice across multiple time points. We identified distinct transcriptional profiles between tumor and normal tissues, resolving six major cell types through integrated dimensionality reduction and pathological annotation. Pseudo-time trajectory analysis revealed increased expression of *MMP11* and *MYL9* in later stages of tumor progression. Analysis of human CRC cohorts from the TCGA database further confirmed that high expression of these genes is associated with advanced clinical stages and promotes tumor proliferation and invasion. Temporal gene expression dynamics indicated enrichment of cancer-related pathways concurrent with suppression of lipid and amino acid metabolism. Notably, genes in the *DEFA* family were significantly upregulated in normal tissues compared to tumor tissues. Functional validation showed that *DEFA3* inhibits colon cancer cell migration and proliferation in vitro. These demonstrate the value of ST in resolving spatiotemporal heterogeneity in CRC and identify both *MMP11*/*MYL9* and *DEFA3* as potential biomarkers and therapeutic targets.

## 1. Introduction

Colorectal cancer (CRC) is the second-most deadly cancer in the world, which occurs in the colorectal mucosa and gland malignant tumors, and accounts for nearly 900,000 annual deaths [1,2]. In recent years, the mainstay treatments for CRC have included minimally invasive techniques for early-stage disease [3,4] and surgical resection for advanced cases [5]. Among the key molecular pathways involved, the WNT signaling pathway plays a critical role. Mutations or inactivation of the *APC* gene can impair the function of the destruction complex, leading to constitutive activation of the WNT pathway [6]. This results in the stabilization and nuclear translocation of β-catenin, where it activates target oncogenes, ultimately promoting glandular epithelial hyperplasia and adenoma formation [6,7]. Despite advances, the molecular mechanisms underlying CRC remain incompletely understood. Recent advances in molecular biology and sequencing technologies have enabled a deeper investigation into colorectal carcinogenesis. However, conventional RNA sequencing approaches are often limited in their ability to capture the complete transcriptomic landscape, as they lack spatial and cellular resolution. In comparison, single-cell sequencing technology has emerged as a powerful tool for characterizing genetic and functional heterogeneity by profiling gene expression at the single-cell level [8,9]. Its application to tumor tissues has provided valuable insights into cancer biology and mechanisms of tumorigenesis. Nevertheless, single-cell sequencing requires tissue dissociation, which disrupts native spatial architecture and loses critical contextual information on the cellular microenvironment. Analyzing location-dependent gene expression patterns is essential for understanding tumor initiation and progression. To address this limitation, spatial transcriptomics (ST) has been developed [10]. ST provides genome-wide expression profiling within the spatial context of intact tissue, offering valuable opportunities to elucidate tumor-related processes. As a result, the application of ST to investigate the dynamics of tumor development has become a major research focus.

## 2. Results

### 2.1. Spatial Transcriptomics Delineates the Cellular Architecture of Colorectal Tissues

**Figure 1:**
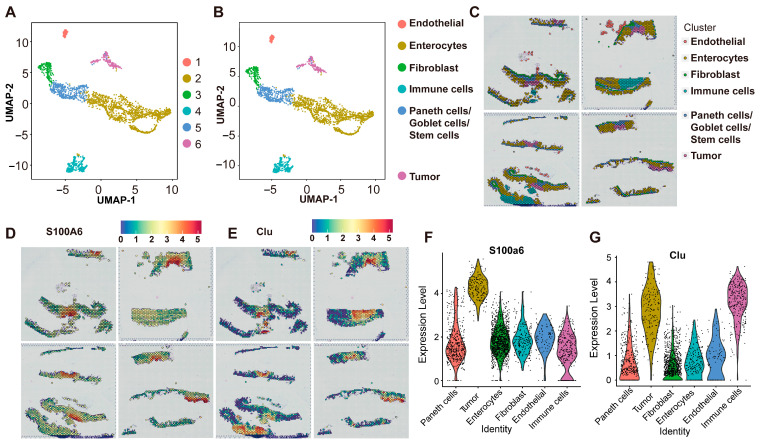
ST to study CRC (*n* = 4): (**A**) UMAP plot of the principal component analysis, first (PCA) clustering the results profiled in the present work; (**B**) UMAP plot of cell types; (**C**) spatial images of unsupervised clustering results; (**D**,**E**) spatial plots showing the spatial expression pattern of S100A6 and CLU; (**F**,**G**) violin plots showing the expression of S100A6 and CLU.

We used spatial transcriptome data from intestinal tumor tissues of *APC ^Min/+^* mice. A total of 2482 capture points were obtained from the slices. We first labeled the locations of tumor tissue and normal tissue (Figure S1A) stained with H&E and then performed cluster analysis on all points and visualized the clusters using the uniform manifold approximation and projection (UMAP) method. All points were aggregated into six clusters (Figure 1A) according to the pathological information of the cell type (Figure S1B), which can be divided into six categories through different cell markers, namely, endothelial cells, epithelial cells, tumor cells, immune cells, and fibroblasts (Figure 1B). Here, we found that unsupervised clustering analysis could cluster ST points with similar characteristics, such as immune cells and tumor regions, and we located various types of cells back on the spatial transcriptome map (Figure 1C). Our analysis results show that S100A6 and CLU exhibit high expression in intestinal tumor tissue (Figure 1D–G).

### 2.2. Gene Changes in the Development of APC^Min/+^ Tumor Tissue over Time

**Figure 2:**
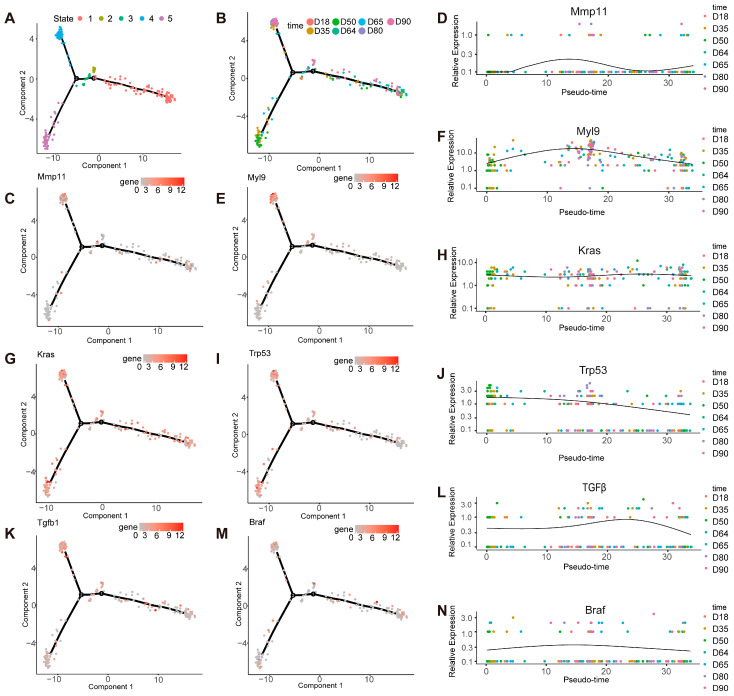
ST discovery of differential genes through pseudo-time analysis (*n* = 4): (**A**,**B**) trajectory of differentiation of the different time points predicted by monocle; (**C**) differential expression of *MMP11* at distinct time points; (**D**) gene expression levels at single spots ordered along the pseudo-time for *MMP11*; (**E**) differential expression of *MYL9* at distinct time points; (**F**) gene expression levels at single spots ordered along the pseudo-time for *MYL9*; (**G**) differential expression of *KRAS* at distinct time points; (**H**) gene expression levels at single spots ordered along the pseudo-time for *KRAS*; (**I**) differential expression of *P53* at distinct time points; (**J**) gene expression levels at single spots ordered along the pseudo-time for *P53*; (**K**) differential expression of transforming growth factor-beta (*TGF-β*) at distinct time points; (**L**) gene expression levels at single spots ordered along the pseudo-time for TGF-β; (**M**) differential expression of *BRAF* at distinct time points; (**N**) gene expression levels at single spots ordered along the pseudo-time for *BRAF*.

We aimed to investigate the progression of *APC ^Min/+^* from tumorigenesis to the stabilization of tumor numbers, spanning from 18 days to 90 days, to describe the changes in tumor tissue. According to the pseudo-time analysis of cell differentiation, we divided it into five states (Figure 2A) and then mapped each state to its corresponding time point. We found that there were two types of differentiation tracks in the intestinal tissue of mice from 18 days to 90 days, one of which was a process of transitioning from 18 days to 80 days along the time axis and, finally, reaching 90 days, and the other was a process of transitioning from 18 days along the event to 35 days and from 50 days to 65 days, based on the results of the time points (Figure 2B). State 4 can be through of as the middle stage and State 5 as the late stage. We identified two genes with high expression in the late stage. The expression level of matrix metalloproteinase-11 (*MMP11*) tends to increase over time (Figure 2C,D). Additionally, the expression level of the myosin light chain 9 (*MYL9*) gene is very high in advanced CRC (Figure 2E,F). We found that the *KRAS* (Figure 2G,H) and *TGF-β* (Figure 2K,L) genes continue to be expressed during differentiation, with relatively high levels of expression, while there is a fluctuation in the expression level of *Tp53* (Figure 2I,J). These results are consistent with the changes in gene expression in tumor tissue prior to the onset of disease. The expression level of the *BRAF* gene is relatively low during the overall differentiation process (Figure 2M,N). We divided all gene expression patterns into four clusters by analyzing gene expression in pseudo-time analysis (Figure S1C).

### 2.3. MMP11 and MYL9 Exploration and Functional Assays Using Publicly Available Datasets

**Figure 3:**
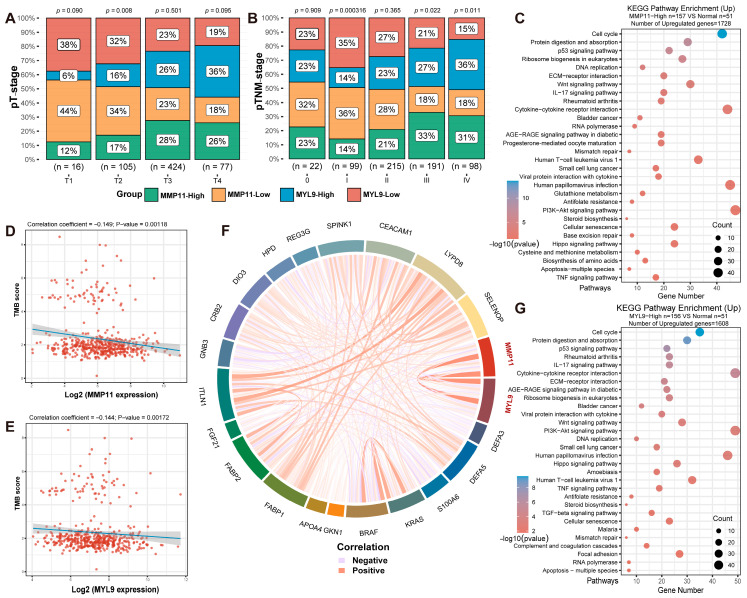
(**A**,**B**) Presents the distribution of tumor (T) and p-TNM staging in different sample groups, with the x-axis representing the different sample groups, the y-axis representing the percentage of clinical information contained in the corresponding grouped samples, and the different colors representing various clinical information. Significance, as a *p*-value, was analyzed by chi-square test, where the numerical size is −log10 (*p*-value). (**C**,**G**) Differentially expressed genes were screened based on samples from the *MMP11* high-expression group (*n* = 157), *MYL9* high-expression group (*n* = 156), and para-tumoral tissue group (*n* = 51), which was followed by KEGG analysis of the upregulated genes. According to the KEGG pathway enrichment results, the different colors represent the significance of the functional enrichment results, with larger values indicating smaller *p*-values. The size of the circle represents the number of enriched genes, with larger circles indicating more genes. (**D**,**E**) Illustration of the Scatter plot and fitted line of the Spearman correlation analysis between TMB and *MMP11* and *MYL9* gene expression. Each point represents a sample. The x-axis represents the distribution of gene expression, and the y-axis represents the score distribution of TMB. The density curve on the bottom depicts the distribution trend of the TMB scores and the gene expression. The top of the figure displays the *p*-value, correlation coefficient, and the method used for correlation calculation. (**F**) Multiple gene Spearman correlation circle plots, where the different colors represent correlation coefficients (red indicates a positive correlation and blue indicates a negative correlation in the diagram), with darker shades denoting stronger correlations among variables.

We downloaded relevant data on CRC patients from the TCGA database, encompassing a total of 620 cases. Subgroup analyses were conducted based on *MMP11* expression levels (*MMP11* high-expression group, *n* = 157; *MMP11* low-expression group, *n* = 157, top 25%) and *MYL9* expression levels (*MYL9* high-expression group, *n* = 156; *MYL9* low-expression group, *n* = 155, top 25%), including a control group of para-tumoral tissue (*n* = 51). We utilized the aforementioned four subgroups to compare differences in clinical p-TNM staging. Specifically, for p-T staging (Figure 3A), p-N staging (Figure S2C), p-M staging (Figure S2D), and p-TNM staging (Figure 3B), the percentage of patients with high *MMP11* and *MYL9* expression significantly increased, with statistical significance observed for p-TNM stages III and IV (*p* < 0.05). Moreover, in tumor tissues from CRC patients, the expression levels of *MMP11* and *MLY9* showed a positive correlation (Figure 3F). We analyzed the relationship between *MMP11* and *MYL9* gene expression and tumor mutational burden. We found that high expression of *MMP11* and *MYL9* genes was negatively correlated with tumor mutation burden (TMB) scores (*p* < 0.05) (Figure 3D,E). By comparing differentially expressed genes between the *MMP11* and *MYL9* high-expression groups and the adjacent non-cancerous tissue group, we found that 1728 genes were upregulated and 1038 genes were downregulated in the *MMP11* high-expression group. In the *MYL9* high-expression group, 1608 genes were upregulated and 891 genes were downregulated. GO analysis indicated that Biological Process terms in both groups clustered around extracellular matrix organization and extracellular structure organization; Molecular Function terms clustered around signaling receptor activator activity and receptor ligand activity; Cellular Component terms clustered around collagen-containing extracellular matrix and endoplasmic reticulum lumen (Figure S3A,B). KEGG pathway enrichment analysis revealed that pathways in both groups were primarily concentrated in the cell cycle, protein digestion and absorption, and p53 signaling pathways (Figure 3C,G).

### 2.4. Temporal Dynamics of Pathway Activation and Suppression During Tumor Progression

We chose to integrate these data for analysis. We took 18 days, 35 days, and 40 days as the pre-tumor stage; 50 days, 64 days, and 65 days as the mid stage of tumor development; and 80 days and 90 days as the late stage of tumor development. We conducted GO and KEGG analyses on these three stages. Based on the analysis results, it was found that biological processes related to DNA, RNA, and ribosomes were activated in the early stages of tumor development, and splicing-related biological processes were also activated. Therefore, splicing is very important for the tumorigenesis process, which requires the synthesis of a large number of proteins. The intestinal absorption function, digestion process, lipid catabolism, and carbohydrate metabolism biological processes were all inhibited (Figure S1D).

**Figure 4:**
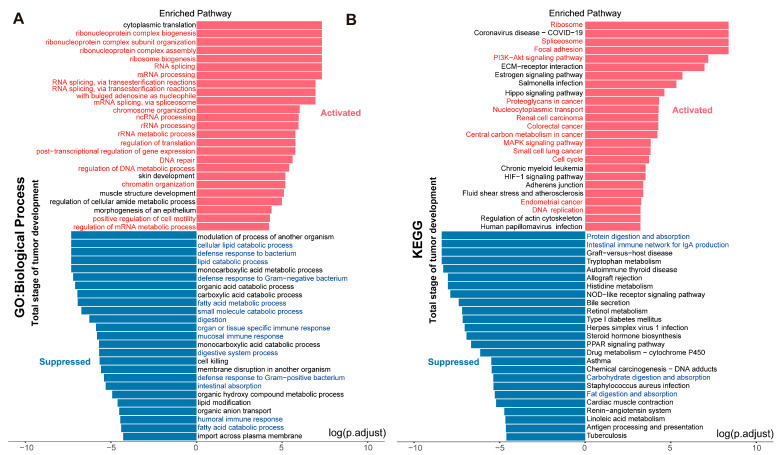
Enrichment analysis of tumor-related pathways: (**A**) GO analysis across all stages of tumor development; (**B**) KEGG analysis across all stages of tumor development. Red indicates activation; blue indicates suppression.

We explored the KEGG pathways during this period (Figure S1F), and the signaling pathways activated in the early stages of tumor development include the ribosome signaling pathway. The pathways suppressed in the later stages of tumor development were largely consistent with those in the previous two stages (Figure S1). We studied the difference in gene expression between tumor tissue and peripheral tissue in more detail. We confirmed the obvious separation between tumor tissue region and normal tissue using PCA (Figure S2A). Then, we integrated all time points, and the integrated results are basically consistent with the activation and inhibition of related biological processes at each time period (Figure 4A). Compared to normal epithelial cells, genes in tumor tissue are enriched in different types of cancer-related pathways, including proteoglycans in cancer, nucleocytoplasmic transport, renal cell carcinoma, CRC, central carbon metabolism in cancer, small-cell lung cancer, and endometrial cancer (Figure 4B).

### 2.5. Spatial and Temporal Analysis Identifies DEFA Family Genes as Potential Tumor Suppressors

**Figure 5:**
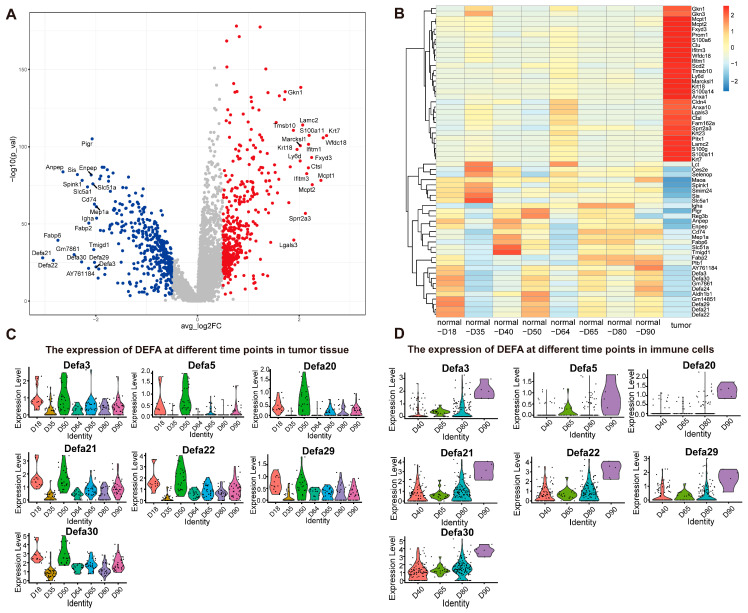
Differentially expressed genes between tumor and normal peripheral tissues: (**A**) volcano plot of significantly differentially expressed genes between tumor and normal peripheral tissues; Each point represents a gene. (**B**) heatmap of the most variable genes between tumor and normal peripheral tissues at different time points; (**C**) violin plots showing the expression of *DEFA* genes at different time points in tumor tissue; (**D**) violin plots showing the expression of *DEFA* genes at different time points in immune cells. Each point in (**C**,**D**) represents a measurement from an organizational spatial unit (capture point).

**Figure 6:**
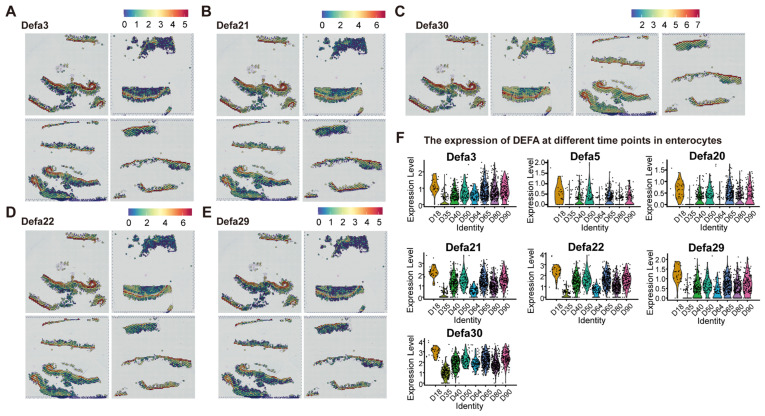
(**A**–**E**) Spatial plots showing the spatial expression patterns of the *DEFA* family; (**F**) violin plots showing the expression of *DEFA* genes at different time points in enterocytes. The dots above the violin plot represent the actual expression measurements of the *DEFA* gene at each specific time point, derived from all individual spatial capture points identified as intestinal epithelial cells.

We integrated data from all points in time. A volcano map and heatmap show that compared with the tumor tissue the expression of *DEFA* genes in the normal tissue increased (Figure 5A,B), including in *DEFA3*, *DEFA21*, *DEFA22*, *DEFA24*, *DEFA29*, and *DEFA30*. We first analyzed the expression of *DEFA* genes in several parts of tissues by a violin diagram. The results show that the expression of *DEFA* genes in tumor tissue increased first and then decreased (Figure 5C). In contrast, the expression of *DEFA* in immune cells increased over time (Figure 5D), while the expression of *DEFA* in Paneth cells did not exhibit a difference over time, which may be because *DEFA* was produced by Paneth cells (Figure S2B). There were also differences in the expression of *DEFA* genes in other types of cells, such as endothelial cells (Figure S2E), fibroblasts (Figure S2F), and enterocytes (Figure 6F). We located these *DEFA* genes on the spatial transcriptome map and found that the expression of *DEFA* in tumor tissue was significantly lower (Figure 6A–E).

We aimed to verify the function of *DEFA* further to determine whether it affects the proliferation, migration, and invasion of CRC cells. We selected the CT26 CRC cell line as the research object. The wound-healing assay showed that, compared with the control group, the addition of DEFA3 in vitro could effectively inhibit the migration of tumor cells (Figure S3C); then, the CCK-8 assay showed that the proliferation of CRC cells treated with *DEFA3* decreased (Figure S3D). Finally, we treated CT26 cells with DEFA3 and used Ki-67 to detect the proliferation for immunofluorescence. Here, we found that the fluorescence intensity of the CT26 cell line treated with *DEFA3* decreased (Figure S3E), which further showed the inhibitory effect of *DEFA3* on tumor cell proliferation. Finally, we conducted a gene network analysis of the *DEFA* gene (Figure S5) and found that there were tumor-suppressor-related genes associated with *DEFA*, including *FGF21* and *ITLN1*, as well as tumor-promoting genes associated with *DEFA*, including *GNB3*, *CRB2*, *DIO3*, *HPD*, and *Dll3*. These results suggest that *DEFA* plays a crucial role in the development and progression of tumors.

## 3. Discussion

Tumor research has advanced in tandem with sequencing technologies, transitioning from bulk sequencing to single-cell sequencing. Single-cell sequencing enables investigation of cellular heterogeneity at a single-cell resolution and has become widely used for detecting tumor genes [11,12]. However, the tissue dissociation step required for single-cell sequencing inevitably disrupts the native spatial architecture of tissues and erases critical contextual information about the cellular microenvironment, limiting efforts to decipher tumor-related mechanisms. ST, named *Nature*’s Method of the Year in 2020 [13], addresses this gap and has since been applied to study a range of diseases. For example, Kuppe et al. employed ST to construct an integrated molecular atlas of spatial gene expression in human myocardial infarction, providing valuable insights for mechanistic and therapeutic research in heart disease [14]. Similarly, Chen et al. employed ST combined with in situ hybridization to reveal multicellular gene co-expression networks in Alzheimer’s disease, two of which were driven by amyloid plaque accumulation [15]. This study has applied spatial transcriptome sequencing to characterize the temporal progression of CRC in *APC ^Min/+^* mice. We constructed a spatial transcriptomic map of CRC development and further defined molecular differences between tumor and normal tissues, as well as differences validated, in part, through known cell markers like S100A6 and CLU. S100A6, a calcium-binding protein of the S100 family, localizes primarily to the cytoplasm of tumor cells [16,17], while CLU, a multifunctional protein, is significantly upregulated under cellular stress in diseases including cancer [18]. Our data confirm their high expression in murine intestinal tumor regions.

A key focus of our work was analyzing intestinal tumors in mice at various time points. This approach allowed us to identify differentially expressed genes in *APC ^Min/+^* mice during the late stages of intestinal tumorigenesis and track how tumor-related gene expression changes over time. Notably, our results show that *MMP11* and *MYL9* are highly expressed in late-stage tumors. *MMP11* serves as a key mediator of normal physiological tissue remodeling, and its upregulation has been previously associated with tumor initiation and malignant progression [19]. Our data confirm its expression increases progressively over time in *APC ^Min/+^* mice. *MYL9*, a fibroblast marker associated with CRC severity, is also highly expressed in advanced CRC [20]. This finding is consistent with our observation of elevated *MYL9* in late-stage murine tumors. Both genes are known to promote tumor proliferation and invasion [19,20], and our spatial transcriptomic data further reinforce their association with intestinal tumor progression. Additionally, we identified several genes associated with canonical colorectal cancer pathways, such as the proto-oncogenes *KRAS* [21] and *BRAF* [22] and the tumor suppressor *TP53* [23], as well as components of the *TGF-β* signaling pathway [23]. *KRAS* and *TGF-β* were consistently expressed throughout differentiation, whereas the expression of *TP53* exhibited dynamic fluctuations—a pattern consistent with previously reported pre-tumor gene expression dynamics [22]. Expression of *BRAF* remained consistently low throughout the process, reflecting its context-dependent role in CRC pathogenesis. We also observed temporal changes in other key genes: lipid metabolism-associated genes such as *APOA4*, *FABP1*, and *FABP2* were upregulated over time [23], which is consistent with reports that excess lipids promote tumor cell proliferation, colonization, and metastasis. In contrast, expression of gastrointestinal factor 1 declined gradually, mirroring its downregulation in gastric cancer tissues [24] and suggesting a conserved role across gastrointestinal malignancies.

The integration of murine spatial transcriptomic data with human CRC datasets, which enhances the translational relevance of our findings [25]. We validated key observations using data from 620 human CRC cases in TCGA database. For instance, in human CRC, high *MMP11* and *MYL9* expression correlated with advanced p-TNM stages (III/IV) and showed a positive correlation with each other—mirroring the temporal upregulation of these genes in late-stage murine tumors. TMB is a critical biomarker for predicting response to immunotherapy [26,27]. Furthermore, our analysis showed that high expression of *MMP11* and *MYL9* in human colorectal cancer was inversely correlated with TMB. This suggests that *MMP11* and *MYL9* may drive CRC progression by contributing to therapy resistance—possibly through reduced tumor immunogenicity or the establishment of an immunosuppressive microenvironment. These findings were consistently observed in both murine and human datasets, supporting the potential of *MMP11* and *MYL9* as clinical biomarkers or therapeutic targets. KEGG analysis also revealed that genes upregulated in human CRC tissues exhibiting high *MMP11*/*MYL9* expression were enriched in key pathways, such as cell cycle and p53 signaling—pathways similarly activated in late-stage murine tumors, strengthening the cross-species relevance of these findings.

To better contextualize these temporal gene expression patterns, we analyzed stage-specific pathway activation and inhibition in *APC ^Min/+^* mice. During the early stages of tumor development, biological processes involving DNA, RNA, and ribosomes were upregulated, along with splicing-related activities. Pre-mRNA splicing removes introns to produce mature mRNA, which then directs protein synthesis during translation [28]. Moreover, this process is critical for tumorigenesis, as cancer cells require massive protein production to sustain their uncontrolled proliferation. Conversely, functions—such as intestinal absorption, digestion, lipid catabolism, and carbohydrate metabolism—were suppressed, which is consistent with established tumor phenotypes in which cancer cells disrupt normal tissue function to prioritize their own survival. For instance, tumor cells can induce lipolysis in adjacent adipocytes to acquire fatty acids for energy production or lipid accumulation, while suppression of cell-killing mechanisms enables unlimited proliferation [29,30,31]. KEGG analysis of early-stage tumors revealed activation of the ribosome signaling pathway, which is critical for synthesizing cellular components required for rapid cell growth, particularly in cancer cells undergoing uncontrolled proliferation [32], as well as the Hippo signaling pathway, which is closely associated with tumor initiation and progression [33]. The estrogen signaling pathway was also activated. Although estrogens promote normal endometrial proliferation, sustained estrogen signaling is a well-established risk factor for endometrial cancer [34]; its activation in early-stage CRC may indicate shared mitogenic mechanisms across cancer types. Additionally, the nucleocytoplasmic transport pathway was activated. In non-tumor cells, APC protein is distributed evenly between the nucleus and cytoplasm, but in approximately 80% of CRC patients, APC mutations result in accumulation of a truncated, stable protein within the nucleus [35], disrupting normal transport mechanisms and promoting WNT pathway dysregulation. The cell cycle pathway was also activated, driven by continuous cyclin-dependent kinase activation via ubiquitin-mediated proteolysis of cyclins and kinase inhibitors [36]. Furthermore, focal adhesions, which regulate directional cell migration [37], were upregulated. The PI3K-Akt pathway, which regulates cell proliferation, differentiation, and survival [38], was activated, as was the TGF-β pathway, with enrichment of tumor-related pathways, including those related to proteoglycans in cancer and renal cell carcinoma. Notably, histidine metabolism was inhibited; recent work shows histidine catabolism enhances methotrexate efficacy by increasing cancer cell sensitivity to the drug [39], suggesting this inhibition may impact chemotherapy responses in early CRC.

In the middle stages of tumor development, enriched biological processes included positive regulation of cell motility, locomotion, and cell division—all of which promote tumor cell proliferation, migration, and invasion. Suppressed processes remained consistent with those observed in the early stages, and KEGG analysis revealed more differentially expressed genes enriched in CRC-specific pathways, such as colon cancer and microRNAs in cancer. The oxidative phosphorylation pathway was inhibited, reflecting the well-documented shift in cancer cells toward glycolysis, which leads to downregulated oxidative phosphorylation in many cancers [40]. In the late stages, activated biological processes mirrored those of earlier stages but with greater inhibition of immune-response-related pathways, likely contributing to immune evasion, as well as more differential genes enriched in diverse tumor-related pathways, such as melanoma and pancreatic cancer. Principal component analysis confirmed clear separation between tumor and normal tissues, and integrated analysis across all time points showed tumor tissues were enriched in cancer-related pathways such as nucleocytoplasmic transport and central carbon metabolism in cancer, which is consistent with human CRC biology.

Also, our study has several limitations that merit detailed discussion. First, the sample size for the murine spatial transcriptomic analyses was relatively small. Larger cohorts of *APC ^Min/+^* mice would help confirm the reproducibility of our temporal gene expression trends and reduce the risk of false-positive or negative findings. Second, the inherent technical constraints of the ST platform used in this study impact data resolution. While we combined ST data with pathological annotation to refine cell type assignments, the lack of a single-cell resolution prevents us from dissecting cell–cell interactions at the individual cell level, which are critical for understanding tumor progression. Third, our functional validation of *DEFA3*, *MMP11*, and *MYL9* was limited to in vitro assays and lacked in vivo confirmation. Although we demonstrated that *DEFA3* inhibits proliferation and migration of CT26 cells in vitro, and linked *MMP11*/*MYL9* expression to advanced CRC in human datasets, we did not test the functional impact of these genes in living tumor models. For example, genetic manipulation of *DEFA3*, *MMP11*, or *MYL9* in *APC ^Min/+^* mice would directly reveal their causal roles in tumor initiation, progression, or metastasis. Additionally, the functional assays for *DEFA3* were limited to a single murine CRC cell line, which may not fully represent the genetic diversity of human CRC. Human CRC subtypes harbor distinct mutations—such as *BRAF V600E*, *NRAS* mutations or mismatch repair deficiency—which can alter cellular responses to regulatory factors like *DEFA3*.

The dual role of the *DEFA* family in cancer warrants even more cautious interpretation in light of our data limitations. Although our findings suggest that *DEFA3* acts as a tumor suppressor in CT26 cells and is downregulated in murine tumors, previous reports have described conflicting roles for other *DEFA* family members. It is worth noting that *DEFA* proteins, which belong to the α-defensin family, are well-established for their broad-spectrum antibacterial and anti-HIV properties [41]. Some studies link *DEFA* family members to pro-tumor effects. *DEFA1-3* are upregulated in lung cancer, renal cell carcinoma, and bladder cancer [42,43], and in bladder cancer, their expression in capillary endothelial cells correlates with in-creased invasiveness [44]. *DEFA* has also been reported to promote tumor cell proliferation in late-stage tumor development [45]. Conversely, *DEFA1* can inhibit angiogenesis by impairing endothelial cell proliferation and migration [46], and high concentrations of *DEFA1-3* exhibit cytotoxicity against cancer cell lines [47], the effect of which was first documented in early studies showing that human and rabbit granulocyte defensins mediate in vitro tumor cell cytolysis. Lower concentrations of *DEFA1-3*, however, may promote cancer cell motility and invasion [48], highlighting the importance of concentration in defining function. In our study, *DEFA* expression in murine immune cells increased progressively over time, whereas in tumor tissues it peaked during early stages before declining, suggesting that the role of *DEFA* may transition from tumor-suppressive to context-dependent or even pro-tumorigenic as the tumor microenvironment evolves. It should also be noted that we did not explore cell-type-specific functions of *DEFA*. For example, *DEFA* produced by Paneth cells—the primary source of intestinal defensins—may exert distinct functional impacts compared to *DEFA* secreted by tumor-infiltrating immune cells. Furthermore, although we identified several *DEFA*-associated genes, such as *FGF21* and *GNB3*, these interactions still require experimental validation. Finally, while *DEFA6* has been proposed as a potential biomarker in CRC, its specificity and sensitivity are demonstrated to be inferior to those of carcinoembryonic antigen [45], underscoring the need for careful interpretation regarding the clinical utility of *DEFA* family members. Further mechanistic studies investigating *DEFA* interactions with signaling components such as bradykinin receptors [49] may provide deeper insight into its context-dependent roles in tumorigenesis.

In summary, our spatial transcriptomic analysis of *APC ^Min/+^* mice reveals critical temporal and spatial dynamics in CRC development and validates key findings in human CRC datasets. We identified *MMP11* and *MYL9* as potential biomarkers for advanced disease stages and underscore the context-dependent role of *DEFA* family genes in tumor progression—supported by stage-specific pathway analyses and cross-species validation. Although this study has limitations, as noted above, addressing them in future work will help elucidate broader mechanistic insights and enhance the translational potential of our findings for targeted therapy development.

## 4. Materials and Methods

### 4.1. Experimental Animals

The control sources for the study include wild-type (WT) mice used in hybridization to generate *APC ^Min/+^* mice, as well as normal tissue regions within the *APC ^Min/+^* mice themselves. The sample size was determined based on literature reviews and prior laboratory research, with strict adherence to the 3R principles. Specifically, the study evaluated tumor development across seven time points—18 days, 35 days, 40 days, 50 days, 64 days, 65 days, 80 days, and 90 days—with 3–5 mice allocated to each time point. To minimize confounding factors, multiple strategies were implemented: all experiments were conducted on weight-matched mice aged 5–6 w, and group allocation was optimized through weight matching and averaging littermates across groups (18–24 g). All experiments were performed using male mice. Blinding was rigorously applied, with both testers and analysts remaining unaware of group allocations throughout the experiment and data analysis.

Housing conditions involved maintaining the mice in a specific pathogen-free (SPF) facility, with ad libitum access to standard animal chow and water. Regular weight monitoring was performed as part of the animal care, with a predefined humane endpoint: euthanasia was administered if a mouse experienced 10% weight loss within one week. Euthanasia for tissue collection was conducted using carbon dioxide asphyxiation. For each time point, representative tumor sites were selected from the euthanized mice for sectioning. Ethical approval for all procedures was granted by the Animal Experimentation Committee of Capital Medical University, with the license number AEEI-2021-083. In addition, the date on which we received approval for an animal ethics code is 3 March 2022. The health status of the mice was ensured by SPF housing, and their genetic background was confirmed as *APC ^Min/+^* mice. The primer sequence is Apc-F: 5′-ATACTACGGTATTGCCCAGC-3′ and Apc-R: 5′-TGTTGTTGGATGGTAAGCAC-3′, and the double band on the agarose gel electrophoresis is *APC ^Min/+^* mice.

### 4.2. Spatial Transcriptomics

#### 4.2.1. Collection and Preparation of Colorectal Cancer Tissues

A small amount of OCT was added to the embedded box and placed in a −80 °C refrigerator. Mice were sacrificed using euthanasia, and the entire intestine was immediately removed and split into the following three parts: jejunum, ileum, and colon. The intestinal contents were rinsed with a mixture of PBS and OCT fluids (PBS: OCT = 1:1). All of the above processes were performed on ice. Subsequently, each part of the intestinal tissue was placed in the frozen tissue-embedding box mentioned above, wrapped in a Swiss roll form, and OCT was added until all intestinal tissues were wrapped and quickly stored in −80 °C refrigerator.

#### 4.2.2. Slide Preparation

Spatial transcriptome sample slides contained four capture regions that could accommodate four individual tissue sections, each with 5000 barcoded spots, at 6.5 × 6.5 mm per slide. Due to the small area of the tumor tissue and the low utilization of slides, we chose to paste two pieces of tissue into a capture area. After rapid staining, if there was tumor tissue in the rapid section, the tumor location on the slide was marked, the mass covered with OCT, and the tissue stored at −80 °C for preservation. The tissue-embedding blocks that formed were, subsequently, attached at a thickness of 10 µm on dedicated slides of the spatial transcriptome. Then, they were incubated for 1 min at 37 °C, fixed in 1% methanol for 5 min, and washed in PBS for 3 min. We applied ST techniques that are currently applicable to all of the slices, yielding conventional hematoxylin–eosin (H&E) stained images, as well as gene expression profiles collected by each microarray spot. Tissue sections were annotated for tumor tissue and normal tissue and were selected.

#### 4.2.3. Tissue Permeabilization

According to the Visium Spatial Tissue optimization slides and kit, provided by 10× Genomics, the tissue permeation conditions were optimized. The main step is to place tissue slices in the seven capture regions of the Visium tissue optimization slide, one of which is set as a positive control without the addition of permutase. Slices were fixed, stained, scanned, and imaged, followed by the addition of translucases, and different penetration times were set. The mRNA released during infiltration can bind to oligonucleotides in the capture region, followed by reverse transcription synthesis of the fluorescent cDNA and imaging. The permeation time with the largest fluorescence signal and the smallest signal diffusion is the best. If the signals are the same, a longer penetration time is the best penetration time.

#### 4.2.4. Visium-Sequencing Library Preparation

We used the Visium spatial gene expression slide and kit, provided by 10× Genomics, to perform spatial gene-expression sequencing. The 10 μM tissue sections were attached to the capture area of the slide. After H&E staining, photos in the open-field mode were taken. The experiment was conducted based on the optimal tissue-permeation time obtained with the tissue permeation slide. Then, reverse-transcription experiments were conducted to obtain cDNA with spatial location information and amplified to build a library.

### 4.3. Spatial-Transcriptomics Data Processing

#### 4.3.1. Quality Control of Sequencing Data and Gene Quantification

Sequencing data were analyzed using Space Ranger (v4.0), an official software package, provided by 10× Genomics, specifically for processing its ST data; raw data generated by high-throughput sequencing were in FASTQ format. Space Ranger first aligned the FASTQ sequencing data to the reference genome, then counted the unique molecular identifiers to avoid a PCR amplification bias, as well as conducted barcode filtering based on the distribution of barcodes (which are used to label spatial positions of capture spots) to, ultimately, generate a gene-barcode expression matrix. After obtaining this matrix, the filtered spot–gene expression matrix was further analyzed using Seurat (v4.0.6), which is capable of integrating gene expression, comparative genomic hybridization arrays, single-nucleotide polymorphism arrays, and clinical data via interactive visualization. Data normalization was performed as the initial step of the downstream analysis [50,51].

#### 4.3.2. Dimensionality Reduction and Clustering

Unsupervised clustering of capture spots was implemented using Seurat software (v4.0.6) [52]. Specifically, linear dimensionality reduction was conducted through PCA based on gene expression levels, and the UMAP dimensionality reduction method was applied to visualize the clustering results of capture spots [53]. This UMAP-based visualization approach preserves the global structural features of the original data to the maximum extent, facilitating clear observation of the separation between different clusters.

#### 4.3.3. Cell Type Annotation and Identification

Cell types corresponding to the results of the dimensionality reduction and clustering were inferred and identified by referencing the descriptions of cell marker genes in the previous literature. Classification was performed using specific marker genes for different cell types, including endothelial cells (*Cdh5*, *Pecam1*, *Cldn5*, and *Eng*), epithelial cells (*Epcam* and *Krt8*), immune cells (*Cd79a*, *Cd79b*, *Cd3d*, *Cd86*, abd *Cd3e*), fibroblasts (*Dcn*, *Thy1*, *Col3a1*, *Col5a2*, and *Col1a2*), Paneth cells (*Pgc*), goblet cells (*Muc2*), stem cells (*Lgr5*), and intestinal tumor cells (*Kras* and *Trp53*) [54,55]. Seurat was used to identify marker genes; genes that were differentially upregulated in each cell cluster relative to other cell clusters were screened out, and these differentially expressed genes were defined as potential marker genes for the corresponding cell type. Finally, dot plots were used to visualize the identified marker genes.

#### 4.3.4. Pseudo-Time Analysis

Pseudo-time analysis was carried out using Monocle 2 (available at http://cole-trapnell-lab.github.io/monocle-release, accessed on 30 May 2023). Prior to conducting Monocle 2 analysis, marker genes were selected from the Seurat clustering results and the filtered raw expression counts of cells, and these marker genes were further used to identify differentially expressed genes that showed expression changes across different clusters, providing a basis for constructing the pseudo-time trajectory of cell differentiation [56].

#### 4.3.5. Gene Enrichment Analysis

Pathway enrichment analysis was performed using the Cluster Profiler package based on the Gene Ontology (GO) and Kyoto Encyclopedia of Genes and Genomes (KEGG) databases. Among them, GO analysis annotated the functions of relevant genes mainly from the following three dimensions: cellular component (CC, describing the subcellular localization of gene products), molecular function (MF, describing the molecular activities of gene products), and biological process (BP, describing the biological processes in which genes participate). The KEGG enrichment analysis annotated the signaling pathways involved in the target genes. For the KEGG analysis, the hypergeometric distribution algorithm was used to calculate the statistical significance of differential gene enrichment in each pathway, with smaller *p*-values indicating stronger associations between the corresponding pathway and the differential genes. A *p*-value < 0.05 was considered statistically significant.

### 4.4. Bioinformatics Analysis of Clinical Human Samples

We downloaded the STAR counts data and the corresponding clinical information for CRC from the TCGA database (https://portal.gdc.cancer.gov). We ultimately selected samples from the *MMP11* high-expression group (*n* = 157), MMP11 low-expression group (*n* = 157), *MYL9* high-expression group (*n* = 156), *MYL9* low-expression group (*n* = 155), and para-tumoral tissue group (*n* = 51) for further analysis. We then extracted data in the TPM format and performed normalization using the log2 (TPM+1) transformation [25], after retaining samples that included both RNA-seq data and clinical information. The data are presented as the mean ± standard deviation. Statistical analysis was performed using R software v4.0.3. A *p*-value < 0.05 was considered statistically significant [25,57]. Functional enrichment analysis included the KEGG pathway enrichment results and GO term enrichment results for the differentially upregulated genes, as well as the KEGG pathway enrichment results and GO term enrichment results for the differentially downregulated genes. These functional enrichment results are derived from the R package Cluster Profiler (v3.18.0) [58,59]. We employed Spearman’s correlation analysis to describe the correlations among quantitative variables that do not follow a normal distribution [60]. We utilized online platforms to complete the relevant bioinformatics analyses (https://www.aclbi.com/static/index.html#/tcga, accessed on 1 September 2025).

### 4.5. Cell Culture

CT26.WT cells were obtained from ATCC (Manassas, VA, USA, CRL-2638) and maintained in RPMI 1640 medium (ATCC, 30-2001). The RPMI 1640 medium was supplemented with 10% fetal bovine serum (FBS, Gibco, Grand Island, NY, USA), 100 U/mL penicillin, and 100 μg/mL streptomycin (Life Technologies, Carlsbad, CA, USA), except as indicated. Cells were grown at 37 °C in a 5% CO_2_ incubator. The CT26.WT cell line was identified with short tandem repeat profiling by the ATCC. Upon receipt from the ATCC, the cells were expanded and, subsequently, stored in liquid nitrogen. The stored vials were thawed for experiments and used in <2 months. All cell lines were confirmed to be negative for mycoplasma by ATCC.

### 4.6. Wound-Healing Assay

The cells were grown as a confluent monolayer in a six-well culture dish. The cell monolayers were scratched, using a sterile p200 pipette tip to create a wound, and then washed with PBS to remove cell debris. The cells were incubated in medium supplemented with culture media with or without DEFA3. Cell migration was monitored under an inverted microscope equipped with a camera. The wound distance (width) at different time points was measured and calculated at 0 h, 24 h, and 48 h.

### 4.7. CCK-8 Assay

Cells were seeded at a density of 3000 per well in 96-well plates and cultured for 24 h in 100 μL medium containing 10% FBS. Next, a 10 μL CCK-8 solution (NCM Biotech, C6005, Suzhou, Jiangsu, China) was added per well, and the cells were cultured at 37 °C. The number of viable cells was evaluated by measuring the absorbance at 450 nm using a SynergyH1 microplate reader (Burlington, VT, USA).

### 4.8. Immunofluorescence

For Immunofluorescence, CT26.WT was processed with PBS or DEFA3 for 12 h. Then, Ki-67 (Cell Signaling Technology, Boston, MA, USA, 12202, dilution 1:400) was used overnight at 4 °C. The secondary antibodies were horseradish-peroxidase-conjugated anti-rabbit IgG (Cell Signaling Technology, Boston, MA, USA, 7074, dilution 1:200) and anti-rabbit IgG (Thermo Fisher Scientific, Waltham, Massachusetts, USA, A-11304, dilution 1:200), incubated at room temperature for 1 h.

## Data Availability

The original contributions presented in this study are included in the article/Supplementary Materials. The ST data generated in this study have been deposited at the China National Center for Bioinformation (https://www.cncb.ac.cn, accessed on 15 September 2025) under project number PRJCA046204. Further inquiries can be directed to the corresponding author.

## Supplementary Materials

The following supporting information can be downloaded at: https://www.mdpi.com/article/10.3390/ijms26189256/s1.

## Abbreviations

The following abbreviations are used in this manuscript:

BRAF	B-Raf Proto-Oncogene
CCK-8	Cell Counting Kit-8
CKIs	Cyclin-Dependent Kinase Inhibitors
CRC	Colorectal Cancer
DEFA	Defensin Alpha
GO	Gene Ontology
H&E	Hematoxylin and Eosin
HIV	Human Immunodeficiency Virus
KEGG	Kyoto Encyclopedia of Genes and Genomes
KRAS	Kirsten Rat Sarcoma Viral Oncogene Homolog
MMP11	Matrix Metallopeptidase 11
mRNA	Messenger RNA
MYL9	Myosin Light Chain 9
PBS	Phosphate-Buffered Saline
PCA	Principal Component Analysis
S100A6	S100 Calcium-Binding Protein A6
ST	Spatial Transcriptomics
TGF-β	Transforming Growth Factor Beta
TMB	Tumor Mutation Burden
UMAP	Uniform Manifold Approximation and Projection
WT	Wild Type
