# Supplementary material for: Investigating the Development of Colorectal Cancer Based on Spatial Transcriptomics
Source: Int J Mol Sci. 2025 Sep 22;26(18):9256. doi: 10.3390/ijms26189256 (PMC12470395; doi:10.3390/ijms26189256)
Supplement: Supplementary file 1 [file ijms-26-09256-s001.zip › ijms-3839651-supplementary.pdf]

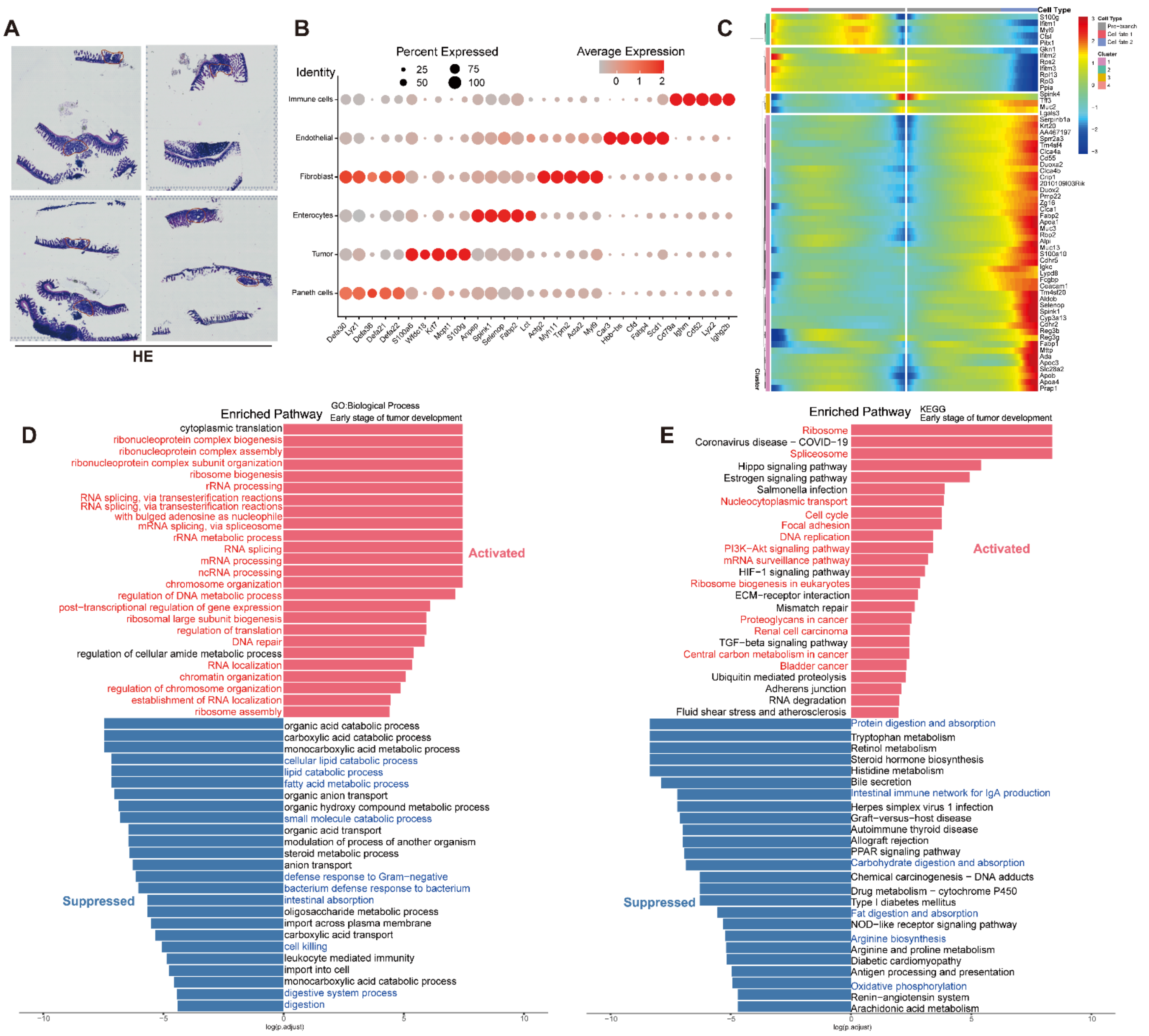

Figure S1 (A) Hematoxylin-eosin staining (H&E) sections annotations. (B) The expression level of cell markers across different morphological regions through the Dot plot. (C) Heatmap shows differential genes through pseudo-time analysis. (D) GO analysis in the early stage of tumor development. (E) KEGG analysis in the early stage of tumor development.

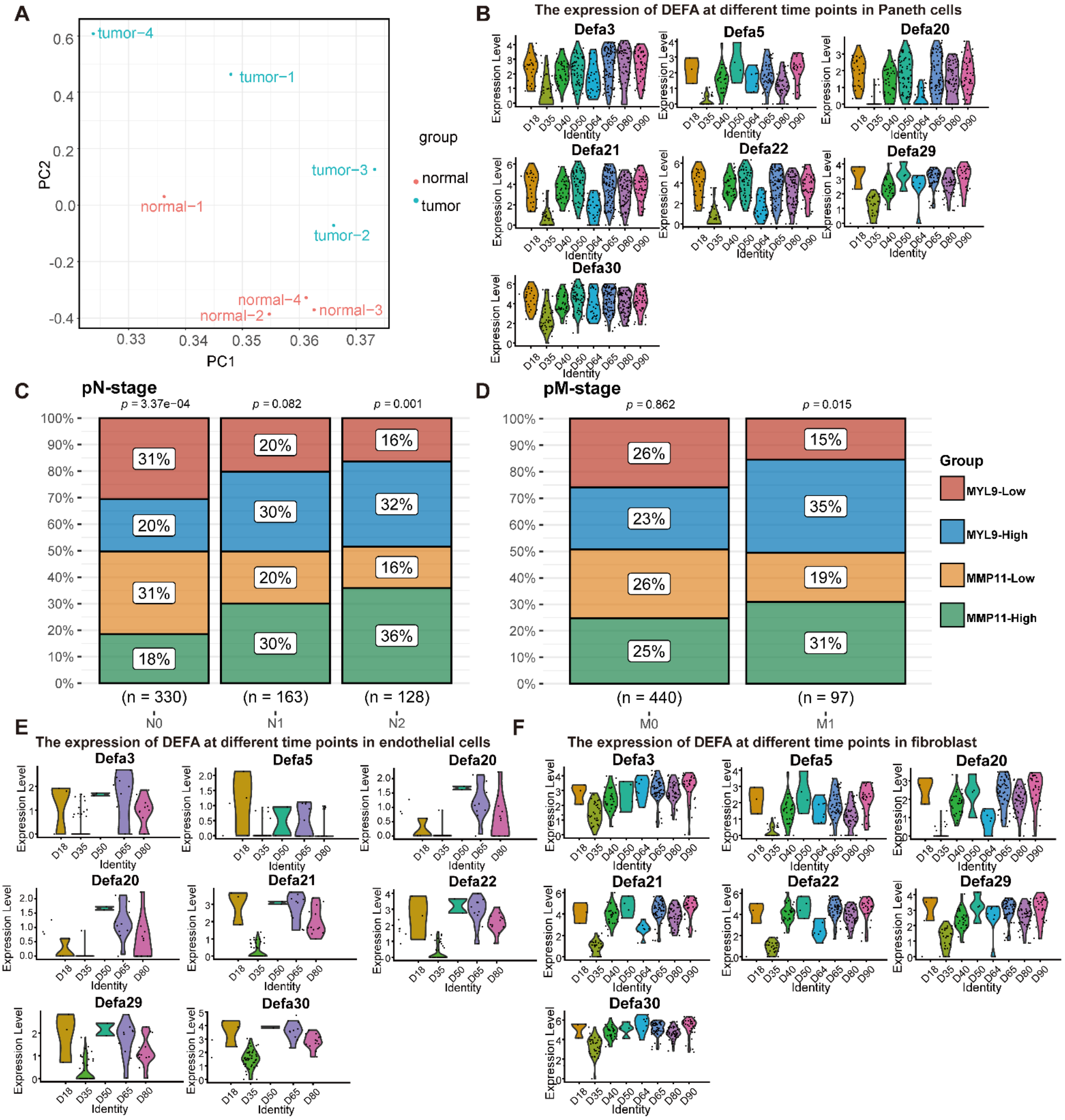

Figure S2 (A) Principal component analysis (PCA) between the two regions. (B) Violin plots show the expression of DEFA gene at different time points in Paneth cells. (C, D) This represents the distribution for lymph nodes (N), and distant metastasis (M) of p-TNM staging in different sample groups, where the x-axis represents different sample groups, the y-axis represents the percentage of clinical information contained in the corresponding grouped samples, and different colors represent different clinical information. And analyzes the significance p-value through the chi-square test, where the numerical size is log10 (P value). (E) Violin plots show the expression of DEFA gene at different time points in endothelial cells. (F) Violin plots show the expression of DEFA gene at different time points in fibroblast.

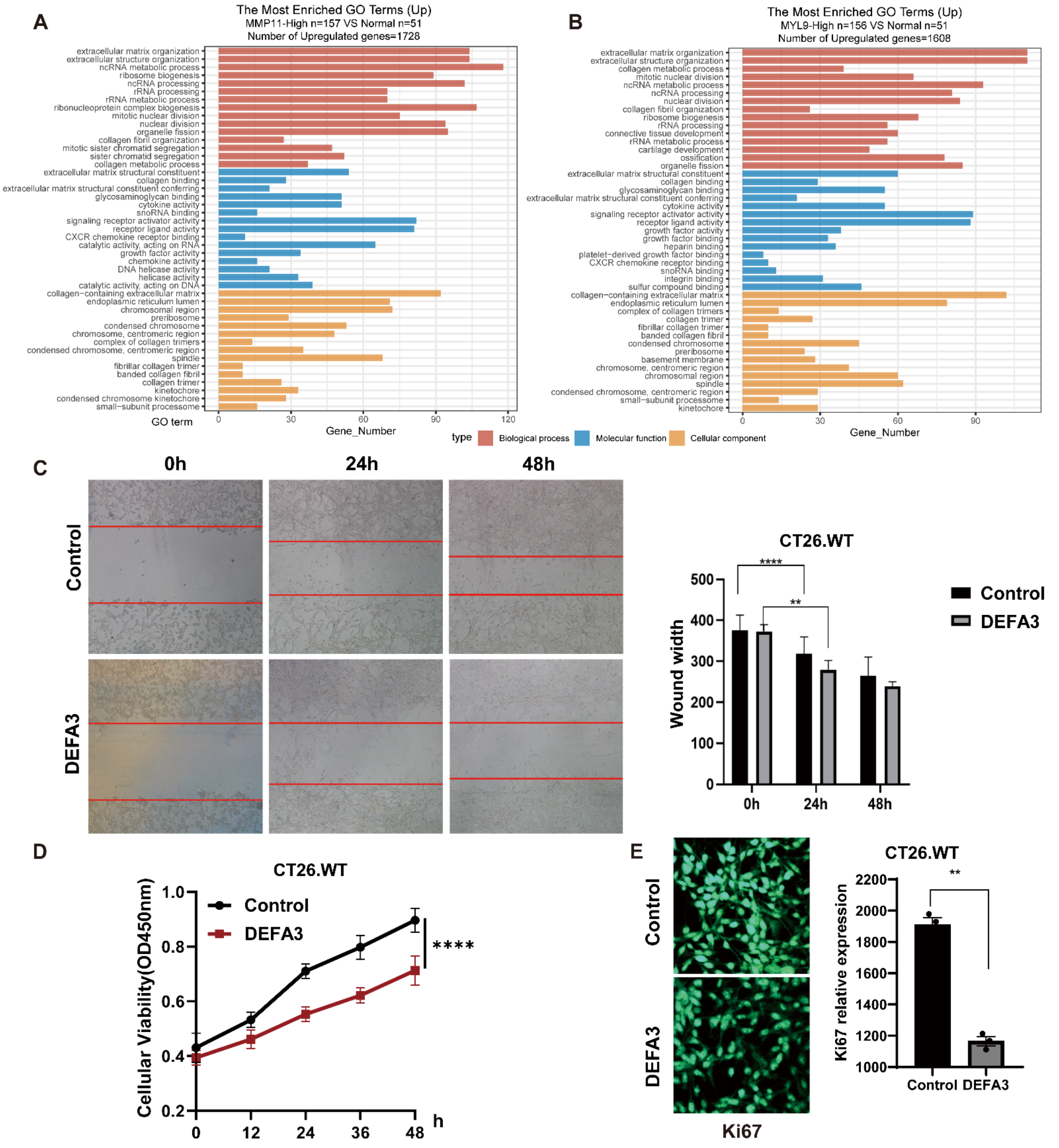

Figure S3 (A, B) Differentially expressed genes were screened based on samples of MMP11 high expression group (n = 157), MYL9 high expression group (n = 156), normal group (n = 51), followed by GO analysis of the upregulated genes. In the GO enrichment function, different colors represent different GO term types, and the length of the bar chart represents the number of genes enriched on the GO term. The chart displays the top 15 most significant results (P < 0.05). DEFA3 inhibits the proliferation and migration of tumor cells. (C) Wound healing assay results for the migration of CT26. WT. (D) CCK-8 assay results for the proliferation of CT26. WT. (E) Immunofluorescence for Ki-67 in CT26.WT treated with PBS or DEFA3.

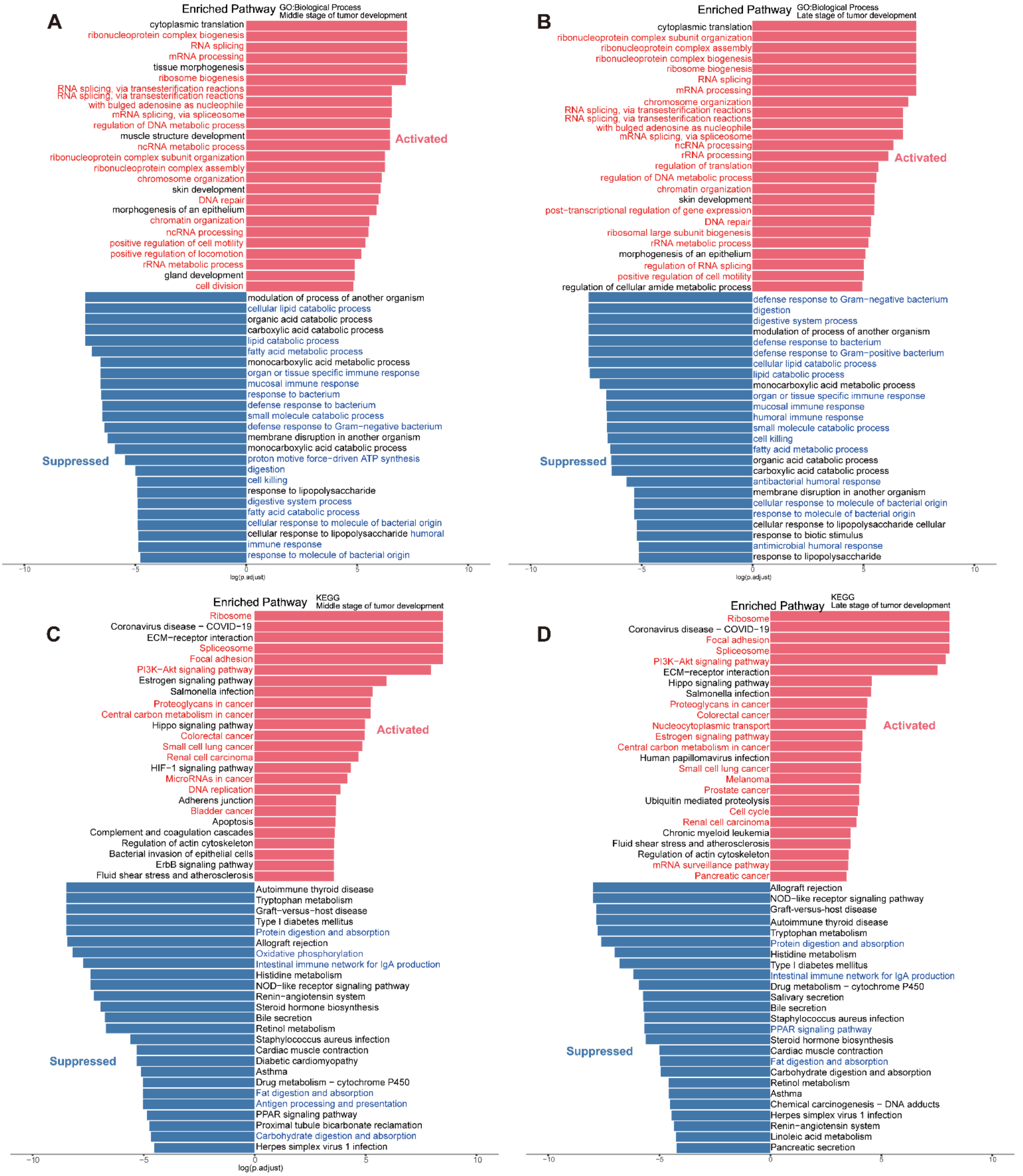

Figure S4 (A, B) GO analysis in the middle and late stage of tumor development. (C, D) KEGG analysis in the middle and late stage of tumor development.

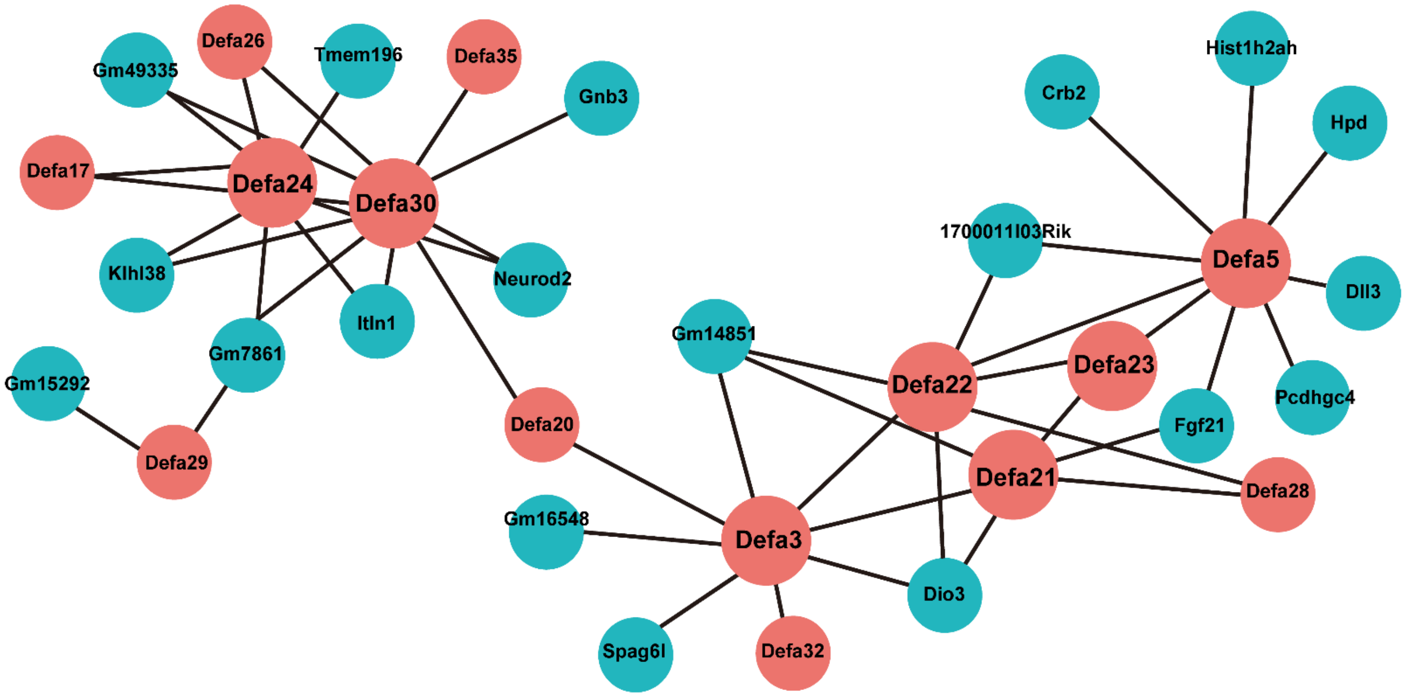

Figure S5 DEFA gene interaction network.
